# Penta­aqua­(5-carb­oxy­pyridine-2-carboxyl­ato-κ^2^ *N*,*O* ^2^)(pyridine-2,5-dicarboxyl­ato-κ^2^ *N*,*O* ^2^)cerium(III) tetra­hydrate

**DOI:** 10.1107/S1600536811052688

**Published:** 2011-12-10

**Authors:** Ning Ma, Tong Zhang, Guang-Rui Yang

**Affiliations:** a Institute of Environmental and Municipal Engineering, North China University of Water Conservancy and Electric Power, Zhengzhou 450011, People’s Republic of China; b Henan Museum, Zhengzhou 450001, People’s Republic of China

## Abstract

In the title compound, [Ce(C_7_H_3_NO_4_)(C_7_H_4_NO_4_)(H_2_O)_5_]·4H_2_O, the Ce^3+^ ion is nine-coordinated by two O atoms and two N atoms from one single and from one double deprotonated pyridine-2,5-dicarboxyl­ate ligand and five water mol­ecules in a distorted monocapped square-anti­prismatic geometry. In the crystal, extensive O—H⋯O hydrogen-bonding inter­actions result in a three-dimensional supra­molecular architecture.

## Related literature

For luminescent lanthanide complexes, see: Faulkner & Pope (2003 ▶). For carb­oxy­lic complexes of lanthanides, see: Cao *et al.* (2002 ▶). For a related europium structure, see: Song *et al.* (2005 ▶). 
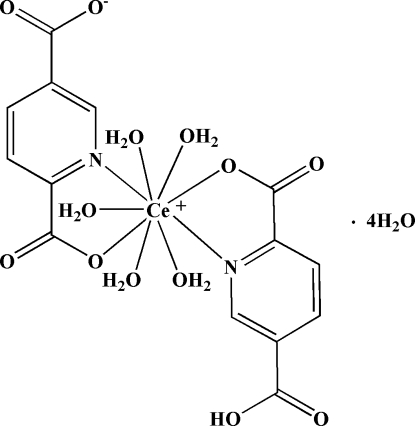


## Experimental

### 

#### Crystal data

[Ce(C_7_H_3_NO_4_)(C_7_H_4_NO_4_)(H_2_O)_5_]·4H_2_O
*M* *_r_* = 633.48
Monoclinic, 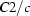

*a* = 14.0652 (10) Å
*b* = 9.6485 (7) Å
*c* = 33.345 (2) Å
β = 93.650 (1)°
*V* = 4516.0 (6) Å^3^
*Z* = 8
Mo *K*α radiation
μ = 2.10 mm^−1^
*T* = 296 K
0.36 × 0.24 × 0.17 mm

#### Data collection

Bruker SMART APEX CCD diffractometer
Absorption correction: multi-scan (*SADABS*; Sheldrick, 2001 ▶) *T*_min_ = 0.518, *T*_max_ = 0.716
11155 measured reflections
3961 independent reflections
3705 reflections with *I* > 2σ(*I*)
*R*_int_ = 0.019

#### Refinement

*R*[*F* ^2^ > 2σ(*F* ^2^)] = 0.021
*wR*(*F* ^2^) = 0.050
*S* = 1.07
3961 reflections
383 parameters
28 restraints
H atoms treated by a mixture of independent and constrained refinement
Δρ_max_ = 0.45 e Å^−3^
Δρ_min_ = −0.50 e Å^−3^

### 

Data collection: *SMART* (Bruker, 2001 ▶); cell refinement: *SAINT-Plus* (Bruker, 2001 ▶); data reduction: *SAINT-Plus*; program(s) used to solve structure: *SHELXS97* (Sheldrick, 2008 ▶); program(s) used to refine structure: *SHELXL97* (Sheldrick, 2008 ▶); molecular graphics: *PLATON* (Spek, 2009 ▶); software used to prepare material for publication: *PLATON*.

## Supplementary Material

Crystal structure: contains datablock(s) global, I. DOI: 10.1107/S1600536811052688/ez2275sup1.cif

Structure factors: contains datablock(s) I. DOI: 10.1107/S1600536811052688/ez2275Isup2.hkl

Additional supplementary materials:  crystallographic information; 3D view; checkCIF report

## Figures and Tables

**Table 1:** Hydrogen-bond geometry (Å, °)

*D*—H⋯*A*	*D*—H	H⋯*A*	*D*⋯*A*	*D*—H⋯*A*
O1*W*—H1*WA*⋯O8*W*	0.83 (1)	1.94 (1)	2.747 (3)	165 (3)
O1*W*—H1*WB*⋯O3^i^	0.83 (1)	1.81 (1)	2.630 (3)	170 (3)
O2*W*—H2*WA*⋯O6*W*	0.83 (1)	1.96 (1)	2.779 (3)	167 (3)
O2*W*—H2*WB*⋯O6*W*^ii^	0.83 (1)	2.11 (2)	2.898 (3)	159 (3)
O3*W*—H3*WA*⋯O7*W*	0.83 (1)	1.98 (1)	2.816 (3)	177 (3)
O3*W*—H3*WB*⋯O4^iii^	0.83 (1)	2.01 (1)	2.823 (3)	165 (4)
O4*W*—H4*WA*⋯O7*W*^iv^	0.83 (1)	1.86 (1)	2.687 (3)	175 (3)
O4*W*—H4*WB*⋯O9*W*	0.83 (1)	1.88 (1)	2.689 (3)	166 (3)
O5*W*—H5*WA*⋯O8^v^	0.83 (1)	1.96 (1)	2.789 (3)	175 (3)
O5*W*—H5*WB*⋯O2^vi^	0.83 (1)	2.01 (1)	2.821 (3)	167 (4)
O6*W*—H6*WA*⋯O2^vi^	0.83 (1)	2.04 (2)	2.823 (3)	157 (3)
O6*W*—H6*WB*⋯O3^vii^	0.83 (1)	1.97 (3)	2.698 (3)	146 (4)
O7*W*—H7*WA*⋯O8*W*^vii^	0.83 (1)	1.94 (1)	2.747 (4)	164 (3)
O7*W*—H7*WB*⋯O6^viii^	0.83 (1)	2.28 (2)	3.054 (3)	157 (5)
O7*W*—H7*WB*⋯O8^v^	0.83 (1)	2.45 (4)	2.898 (3)	115 (3)
O8*W*—H8*WA*⋯O2^ix^	0.82 (1)	2.00 (2)	2.765 (3)	155 (3)
O8*W*—H8*WA*⋯O1^ix^	0.82 (1)	2.64 (2)	3.364 (3)	148 (3)
O8*W*—H8*WB*⋯O7^ix^	0.83 (1)	2.12 (2)	2.901 (3)	158 (4)
O9*W*—H9*WA*⋯O4^x^	0.83 (1)	1.94 (1)	2.750 (3)	167 (4)
O9*W*—H9*WB*⋯O5*W*^xi^	0.82 (1)	2.33 (2)	3.087 (4)	154 (4)
O7—H7⋯O6^xii^	0.82 (1)	1.71 (1)	2.526 (3)	173 (5)

Symmetry codes: (i) 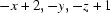; (ii) 
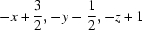
; (iii) 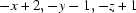; (iv) 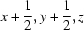; (v) 
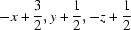
; (vi) 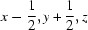; (vii) 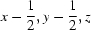; (viii) 
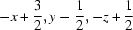
; (ix) 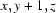; (x) 
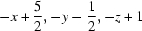
; (xi) 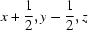; (xii) 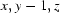.

**Fig. 1.**
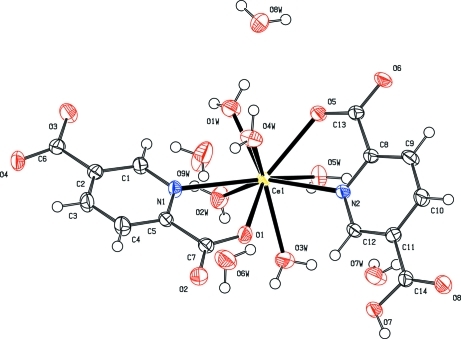
The molecular structure of (I), showing the atom-labelling scheme. Displacement ellipsoids are drawn at the 50% probability level.

**Fig. 2.**
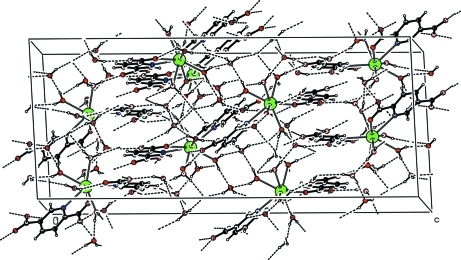
Three-dimensional structure of (I) by means of hydrogen bonds shown as dashed lines. Displacement ellipsoids are drawn at the 50% probability level.

## supplementary crystallographic information

### Comment

The design and synthesis of luminescent lanthanide complexes have been attracting chemists' interest, because of their interesting photophysical properties and their potential application in sensors, optical fiber lasers and amplifiers, luminescent labels for biomolecular interactions, and as electroluminescent materials (Faulkner & Pope, 2003). During the past years, lots of such lanthanide compounds based on multifunctional carboxylic acid ligands have been reported (Cao *et al.*, 2002). Herein, we report the title compound (I).

The title complex, Ce(C_7_H_4_NO_4_)(C_7_H_3_NO_4_)(H_2_O)_5_^.^4(H_2_O), presents a mononuclear molecular structure, which contains a Ce(C_7_H_4_NO_4_)(C_7_H_3_NO_4_)(H_2_O)_5_ molecule and four water molecules (Fig.1), and which is isostructural with its europium analogue (Song *et al.*, 2005). In the molecular structure, the Ce atom resides in a distorted square antiprism geometry, which is defined by two oxygen atoms and two nitrogen atoms from two 2,5-pyridinedicarboxylate ligands and five water molecules. Two oxygen atoms from the two 2,5-pyridinedicarboxylate anions have bond distances of 2.4566 (18) Å [Ce(1)–O(1)] and 2.5098 (18) Å [Ce(1)–O(5)]. Two pyridine nitrogen atoms from the two dinic molecules have bond distances of 2.710 (2) Å [Ce(1)–N(1)] and 2.695 (2) Å [Ce(1)–N(2)]. Five oxygen atoms of the coordinated water molecules have Ce–O distances ranging from 2.449 (2) to 2.573 (2) Å. There are two 2,5-pyridinedicarboxylate anions per molecular unit: one is dianionic and the other must be monoprotonated to maintain electroneutrality.

In addition, the presence of many water molecules in the complex leads to numerous O—H···O hydrogen-bonding interactions including intra- and inter- hydrogen bonds (Fig.2, Table 1), which consolidate a stacked arrangement resulting in a three-dimensional supramolecular architecture.

### Experimental

All reagents were commercially available and of analytical grade. The mixture of Ce(NO_3_)_3_.6H_2_O (0.25 mmol, 0.109 g) and 2,5-pyridinedicarboxylic acid (0.5 mmol, 0.084 g) was dissolved in 20 ml H_2_O, and the mixture was adjusted to pH = 4.5 using NaOH solution. The mixture was stirred and refluxed at 80 °C for one hour. The resulting solution was filtered and the filtrate evaporated at room temperature. Two weeks later, colorless block crystals of (I) were obtained.

### Refinement

The H atoms bonded to water were located in a difference synthesis and refined with distance restraints of O—H = 0.83 (1) Å and H···H = 1.37 (2) Å. The hydroxy H atom (H7) was located in a difference Fourier map and refined with an O—H distance of 0.82 (1) Å. All the remaining H atoms were positioned geometrically, with C—H = 0.93 Å, and were refined as riding with *U*_iso_(H) = 1.2*U*_eq_(C).

### Crystal data

**Table:**

[Ce(C_7_H_3_NO_4_)(C_7_H_4_NO_4_)(H_2_O)_5_]·4H_2_O	*F*(000) = 2536
*M**_r_* = 633.48	*D*_x_ = 1.863 Mg m^−^^3^
Monoclinic, *C*2/*c*	Mo *K*α radiation, λ = 0.71073 Å
Hall symbol: -C 2yc	Cell parameters from 2168 reflections
*a* = 14.0652 (10) Å	θ = 2.6–22.7°
*b* = 9.6485 (7) Å	µ = 2.10 mm^−^^1^
*c* = 33.345 (2) Å	*T* = 296 K
β = 93.650 (1)°	Block, colorless
*V* = 4516.0 (6) Å^3^	0.36 × 0.24 × 0.17 mm
*Z* = 8	

### Data collection

**Table:**

Bruker SMART APEX CCD diffractometer	3961 independent reflections
Radiation source: fine-focus sealed tube	3705 reflections with *I* > 2σ(*I*)
graphite	*R*_int_ = 0.019
phi and ω scans	θ_max_ = 25.0°, θ_min_ = 2.5°
Absorption correction: multi-scan (*SADABS*; Sheldrick, 2001)	*h* = −16→12
*T*_min_ = 0.518, *T*_max_ = 0.716	*k* = −11→11
11155 measured reflections	*l* = −38→39

### Refinement

**Table:**

Refinement on *F*^2^	Primary atom site location: structure-invariant direct methods
Least-squares matrix: full	Secondary atom site location: difference Fourier map
*R*[*F*^2^ > 2σ(*F*^2^)] = 0.021	Hydrogen site location: inferred from neighbouring sites
*wR*(*F*^2^) = 0.050	H atoms treated by a mixture of independent and constrained refinement
*S* = 1.07	*w* = 1/[σ^2^(*F*_o_^2^) + (0.0224*P*)^2^ + 6.9052*P*] where *P* = (*F*_o_^2^ + 2*F*_c_^2^)/3
3961 reflections	(Δ/σ)_max_ = 0.002
383 parameters	Δρ_max_ = 0.45 e Å^−^^3^
28 restraints	Δρ_min_ = −0.50 e Å^−^^3^

### Special details

**Table:**

Geometry. All e.s.d.'s (except the e.s.d. in the dihedral angle between two l.s. planes) are estimated using the full covariance matrix. The cell e.s.d.'s are taken into account individually in the estimation of e.s.d.'s in distances, angles and torsion angles; correlations between e.s.d.'s in cell parameters are only used when they are defined by crystal symmetry. An approximate (isotropic) treatment of cell e.s.d.'s is used for estimating e.s.d.'s involving l.s. planes.
Refinement. Refinement of *F*^2^ against ALL reflections. The weighted *R*-factor *wR* and goodness of fit *S* are based on *F*^2^, conventional *R*-factors *R* are based on *F*, with *F* set to zero for negative *F*^2^. The threshold expression of *F*^2^ > σ(*F*^2^) is used only for calculating *R*-factors(gt) *etc*. and is not relevant to the choice of reflections for refinement. *R*-factors based on *F*^2^ are statistically about twice as large as those based on *F*, and *R*- factors based on ALL data will be even larger.

### Fractional atomic coordinates and isotropic or equivalent isotropic displacement parameters (Å^2^)

**Table:**

	*x*	*y*	*z*	*U*_iso_*/*U*_eq_	
Ce1	0.894634 (10)	−0.318396 (14)	0.381434 (4)	0.01818 (6)	
O1	0.97858 (13)	−0.54167 (19)	0.38596 (5)	0.0277 (4)	
O2	1.07372 (15)	−0.70191 (18)	0.41446 (6)	0.0304 (4)	
O3	1.10159 (16)	−0.0673 (2)	0.53150 (6)	0.0372 (5)	
O4	1.17536 (16)	−0.2284 (2)	0.56975 (6)	0.0370 (5)	
O5	0.88653 (14)	−0.15016 (18)	0.32391 (5)	0.0285 (4)	
O6	0.87079 (16)	−0.08997 (19)	0.25957 (5)	0.0357 (5)	
O7	0.88037 (18)	−0.8380 (2)	0.27999 (6)	0.0374 (5)	
O8	0.85018 (17)	−0.79869 (19)	0.21507 (6)	0.0385 (5)	
N1	1.02240 (15)	−0.3588 (2)	0.44467 (6)	0.0211 (5)	
N2	0.87500 (15)	−0.4203 (2)	0.30610 (6)	0.0212 (5)	
C1	1.04597 (19)	−0.2665 (3)	0.47348 (8)	0.0235 (6)	
H1A	1.0159	−0.1805	0.4724	0.028*	
C2	1.11345 (19)	−0.2927 (3)	0.50507 (8)	0.0229 (6)	
C3	1.1603 (2)	−0.4173 (3)	0.50519 (8)	0.0295 (6)	
H3A	1.2068	−0.4375	0.5254	0.035*	
C4	1.1381 (2)	−0.5126 (3)	0.47515 (8)	0.0300 (6)	
H4A	1.1702	−0.5968	0.4746	0.036*	
C5	1.06761 (18)	−0.4811 (3)	0.44591 (7)	0.0202 (5)	
C6	1.1322 (2)	−0.1883 (3)	0.53826 (8)	0.0253 (6)	
C7	1.03803 (18)	−0.5827 (3)	0.41309 (7)	0.0219 (6)	
C8	0.87469 (19)	−0.3296 (3)	0.27554 (8)	0.0209 (5)	
C9	0.8724 (2)	−0.3706 (3)	0.23619 (8)	0.0307 (7)	
H9A	0.8722	−0.3052	0.2157	0.037*	
C10	0.8705 (2)	−0.5100 (3)	0.22733 (8)	0.0316 (7)	
H10A	0.8691	−0.5399	0.2008	0.038*	
C11	0.87054 (19)	−0.6052 (3)	0.25841 (8)	0.0231 (6)	
C12	0.87281 (18)	−0.5545 (3)	0.29736 (7)	0.0228 (6)	
H12A	0.8728	−0.6177	0.3184	0.027*	
C13	0.87783 (19)	−0.1775 (3)	0.28743 (8)	0.0222 (6)	
C14	0.86649 (19)	−0.7566 (3)	0.24921 (8)	0.0237 (6)	
O1W	0.92616 (16)	−0.0802 (2)	0.40398 (6)	0.0348 (5)	
H1WA	0.939 (2)	−0.024 (2)	0.3864 (6)	0.039 (10)*	
H1WB	0.911 (2)	−0.037 (3)	0.4241 (6)	0.049 (10)*	
O2W	0.81108 (15)	−0.2775 (2)	0.44579 (6)	0.0351 (5)	
H2WA	0.7607 (14)	−0.313 (3)	0.4527 (9)	0.048 (11)*	
H2WB	0.837 (2)	−0.235 (4)	0.4650 (8)	0.074 (14)*	
O3W	0.78056 (16)	−0.5204 (2)	0.38981 (7)	0.0369 (5)	
H3WA	0.7345 (15)	−0.528 (3)	0.3730 (7)	0.041 (10)*	
H3WB	0.796 (2)	−0.5992 (18)	0.3977 (10)	0.060 (12)*	
O4W	1.05648 (15)	−0.2898 (2)	0.35667 (7)	0.0334 (5)	
H4WA	1.076 (2)	−0.2140 (18)	0.3486 (10)	0.042 (10)*	
H4WB	1.1030 (15)	−0.339 (3)	0.3637 (11)	0.052 (11)*	
O5W	0.72275 (14)	−0.2396 (2)	0.36277 (6)	0.0329 (5)	
H5WA	0.6985 (19)	−0.254 (4)	0.3399 (4)	0.044 (10)*	
H5WB	0.6823 (18)	−0.240 (4)	0.3798 (7)	0.056 (12)*	
O6W	0.64591 (16)	−0.3678 (3)	0.47916 (7)	0.0428 (5)	
H6WA	0.611 (2)	−0.333 (3)	0.4610 (9)	0.065 (13)*	
H6WB	0.626 (3)	−0.444 (2)	0.4860 (13)	0.099 (18)*	
O7W	0.62864 (17)	−0.5498 (2)	0.33125 (8)	0.0420 (5)	
H7WA	0.5957 (19)	−0.486 (2)	0.3394 (8)	0.034 (10)*	
H7WB	0.639 (3)	−0.539 (4)	0.3073 (5)	0.104 (19)*	
O8W	0.99039 (18)	0.1264 (2)	0.35587 (7)	0.0412 (5)	
H8WA	1.001 (3)	0.192 (2)	0.3712 (9)	0.061 (13)*	
H8WB	0.948 (2)	0.143 (3)	0.3385 (8)	0.060 (13)*	
O9W	1.22355 (19)	−0.4211 (3)	0.37170 (9)	0.0614 (8)	
H9WA	1.253 (2)	−0.387 (3)	0.3916 (7)	0.057 (12)*	
H9WB	1.242 (3)	−0.5001 (19)	0.3673 (11)	0.076 (15)*	
H7	0.872 (3)	−0.9185 (19)	0.2726 (13)	0.092 (16)*	

### Atomic displacement parameters (Å^2^)

**Table:**

	*U*^11^	*U*^22^	*U*^33^	*U*^12^	*U*^13^	*U*^23^
Ce1	0.02396 (9)	0.01492 (9)	0.01536 (9)	0.00190 (6)	−0.00097 (6)	−0.00118 (6)
O1	0.0359 (11)	0.0211 (9)	0.0247 (10)	0.0035 (8)	−0.0088 (8)	−0.0039 (8)
O2	0.0415 (12)	0.0176 (10)	0.0313 (11)	0.0075 (8)	−0.0046 (9)	−0.0043 (8)
O3	0.0661 (15)	0.0216 (11)	0.0231 (10)	0.0080 (10)	−0.0039 (10)	−0.0027 (8)
O4	0.0574 (14)	0.0276 (11)	0.0237 (11)	0.0007 (10)	−0.0157 (10)	−0.0027 (9)
O5	0.0482 (12)	0.0171 (9)	0.0197 (10)	−0.0011 (8)	−0.0015 (9)	−0.0011 (8)
O6	0.0706 (15)	0.0152 (9)	0.0207 (10)	−0.0017 (9)	−0.0025 (10)	0.0026 (8)
O7	0.0731 (16)	0.0152 (10)	0.0222 (11)	−0.0016 (10)	−0.0101 (10)	−0.0005 (9)
O8	0.0713 (16)	0.0210 (11)	0.0220 (11)	0.0018 (10)	−0.0055 (10)	−0.0049 (8)
N1	0.0262 (12)	0.0199 (11)	0.0167 (11)	0.0053 (9)	−0.0028 (9)	−0.0024 (9)
N2	0.0299 (12)	0.0148 (11)	0.0186 (11)	0.0009 (9)	−0.0013 (9)	−0.0001 (9)
C1	0.0297 (15)	0.0191 (13)	0.0212 (13)	0.0062 (11)	−0.0021 (11)	−0.0022 (11)
C2	0.0283 (14)	0.0203 (13)	0.0202 (13)	−0.0014 (11)	0.0015 (11)	−0.0005 (11)
C3	0.0358 (16)	0.0270 (15)	0.0242 (14)	0.0074 (12)	−0.0102 (12)	−0.0016 (12)
C4	0.0383 (16)	0.0230 (15)	0.0276 (15)	0.0116 (12)	−0.0059 (12)	−0.0025 (12)
C5	0.0251 (13)	0.0184 (13)	0.0171 (13)	0.0015 (10)	0.0011 (10)	0.0012 (10)
C6	0.0333 (15)	0.0220 (14)	0.0205 (14)	−0.0038 (12)	0.0018 (12)	−0.0008 (11)
C7	0.0264 (14)	0.0200 (14)	0.0194 (13)	0.0008 (11)	0.0027 (11)	0.0010 (11)
C8	0.0256 (14)	0.0166 (13)	0.0205 (13)	0.0003 (10)	0.0002 (11)	0.0011 (10)
C9	0.0567 (19)	0.0186 (14)	0.0170 (14)	−0.0001 (13)	0.0035 (13)	0.0031 (11)
C10	0.0559 (19)	0.0238 (15)	0.0150 (13)	−0.0010 (13)	0.0005 (13)	−0.0034 (11)
C11	0.0299 (14)	0.0178 (13)	0.0215 (13)	0.0015 (11)	−0.0003 (11)	−0.0020 (11)
C12	0.0338 (15)	0.0164 (13)	0.0179 (13)	0.0007 (11)	−0.0006 (11)	0.0029 (10)
C13	0.0273 (14)	0.0163 (13)	0.0227 (14)	0.0012 (10)	0.0001 (11)	0.0001 (11)
C14	0.0291 (14)	0.0213 (14)	0.0203 (14)	0.0008 (11)	−0.0022 (11)	−0.0008 (11)
O1W	0.0619 (14)	0.0211 (10)	0.0218 (11)	−0.0011 (10)	0.0059 (10)	−0.0045 (9)
O2W	0.0323 (12)	0.0480 (14)	0.0254 (11)	−0.0050 (10)	0.0057 (9)	−0.0072 (10)
O3W	0.0375 (13)	0.0286 (12)	0.0431 (13)	−0.0053 (10)	−0.0086 (10)	0.0091 (10)
O4W	0.0310 (12)	0.0286 (12)	0.0412 (12)	0.0020 (10)	0.0078 (10)	0.0096 (10)
O5W	0.0303 (11)	0.0440 (13)	0.0238 (11)	0.0048 (10)	−0.0025 (9)	−0.0030 (10)
O6W	0.0406 (13)	0.0395 (14)	0.0476 (15)	0.0021 (11)	−0.0033 (11)	0.0162 (12)
O7W	0.0461 (14)	0.0355 (13)	0.0439 (14)	−0.0044 (11)	−0.0002 (11)	0.0143 (11)
O8W	0.0617 (16)	0.0291 (12)	0.0316 (12)	−0.0064 (11)	−0.0068 (11)	−0.0066 (10)
O9W	0.0550 (16)	0.0467 (16)	0.078 (2)	0.0185 (13)	−0.0337 (14)	−0.0271 (15)

### Geometric parameters (Å, °)

**Table:**

Ce1—O1W	2.4493 (19)	C4—H4A	0.9300
Ce1—O1	2.4566 (18)	C5—C7	1.508 (4)
Ce1—O4W	2.486 (2)	C8—C9	1.369 (4)
Ce1—O5	2.5098 (18)	C8—C13	1.520 (3)
Ce1—O2W	2.542 (2)	C9—C10	1.377 (4)
Ce1—O3W	2.551 (2)	C9—H9A	0.9300
Ce1—O5W	2.573 (2)	C10—C11	1.384 (4)
Ce1—N2	2.695 (2)	C10—H10A	0.9300
Ce1—N1	2.710 (2)	C11—C12	1.386 (4)
O1—C7	1.256 (3)	C11—C14	1.493 (4)
O2—C7	1.254 (3)	C12—H12A	0.9300
O3—C6	1.259 (3)	O1W—H1WA	0.828 (10)
O4—C6	1.241 (3)	O1W—H1WB	0.830 (10)
O5—C13	1.243 (3)	O2W—H2WA	0.832 (10)
O6—C13	1.255 (3)	O2W—H2WB	0.829 (10)
O7—C14	1.297 (3)	O3W—H3WA	0.833 (10)
O7—H7	0.820 (10)	O3W—H3WB	0.831 (10)
O8—C14	1.217 (3)	O4W—H4WA	0.830 (10)
N1—C1	1.337 (3)	O4W—H4WB	0.828 (10)
N1—C5	1.340 (3)	O5W—H5WA	0.828 (10)
N2—C12	1.327 (3)	O5W—H5WB	0.829 (10)
N2—C8	1.343 (3)	O6W—H6WA	0.826 (10)
C1—C2	1.395 (4)	O6W—H6WB	0.827 (10)
C1—H1A	0.9300	O7W—H7WA	0.825 (10)
C2—C3	1.370 (4)	O7W—H7WB	0.826 (10)
C2—C6	1.508 (4)	O8W—H8WA	0.824 (10)
C3—C4	1.381 (4)	O8W—H8WB	0.825 (10)
C3—H3A	0.9300	O9W—H9WA	0.825 (10)
C4—C5	1.380 (4)	O9W—H9WB	0.821 (10)
			
O1W—Ce1—O1	136.63 (7)	C4—C3—H3A	120.1
O1W—Ce1—O4W	81.14 (7)	C5—C4—C3	119.0 (2)
O1—Ce1—O4W	70.80 (7)	C5—C4—H4A	120.5
O1W—Ce1—O5	68.06 (6)	C3—C4—H4A	120.5
O1—Ce1—O5	127.76 (6)	N1—C5—C4	122.3 (2)
O4W—Ce1—O5	70.87 (7)	N1—C5—C7	116.2 (2)
O1W—Ce1—O2W	71.32 (7)	C4—C5—C7	121.5 (2)
O1—Ce1—O2W	109.29 (7)	O4—C6—O3	125.8 (3)
O4W—Ce1—O2W	138.20 (7)	O4—C6—C2	117.7 (2)
O5—Ce1—O2W	122.95 (7)	O3—C6—C2	116.5 (2)
O1W—Ce1—O3W	141.81 (7)	O2—C7—O1	124.2 (2)
O1—Ce1—O3W	68.07 (7)	O2—C7—C5	118.6 (2)
O4W—Ce1—O3W	135.80 (7)	O1—C7—C5	117.2 (2)
O5—Ce1—O3W	125.42 (7)	N2—C8—C9	122.5 (2)
O2W—Ce1—O3W	72.45 (7)	N2—C8—C13	115.6 (2)
O1W—Ce1—O5W	86.90 (7)	C9—C8—C13	121.8 (2)
O1—Ce1—O5W	135.61 (7)	C8—C9—C10	119.1 (2)
O4W—Ce1—O5W	138.86 (7)	C8—C9—H9A	120.4
O5—Ce1—O5W	68.15 (7)	C10—C9—H9A	120.4
O2W—Ce1—O5W	71.37 (7)	C9—C10—C11	119.2 (2)
O3W—Ce1—O5W	70.39 (7)	C9—C10—H10A	120.4
O1W—Ce1—N2	129.35 (6)	C11—C10—H10A	120.4
O1—Ce1—N2	75.98 (6)	C10—C11—C12	117.8 (2)
O4W—Ce1—N2	76.86 (7)	C10—C11—C14	119.8 (2)
O5—Ce1—N2	61.75 (6)	C12—C11—C14	122.4 (2)
O2W—Ce1—N2	144.87 (7)	N2—C12—C11	123.3 (2)
O3W—Ce1—N2	78.20 (7)	N2—C12—H12A	118.4
O5W—Ce1—N2	80.99 (6)	C11—C12—H12A	118.4
O1W—Ce1—N1	78.35 (7)	O5—C13—O6	125.5 (2)
O1—Ce1—N1	62.21 (6)	O5—C13—C8	117.3 (2)
O4W—Ce1—N1	72.46 (7)	O6—C13—C8	117.2 (2)
O5—Ce1—N1	133.10 (7)	O8—C14—O7	123.3 (2)
O2W—Ce1—N1	71.63 (7)	O8—C14—C11	121.4 (2)
O3W—Ce1—N1	101.26 (7)	O7—C14—C11	115.3 (2)
O5W—Ce1—N1	142.85 (6)	Ce1—O1W—H1WA	116 (2)
N2—Ce1—N1	134.12 (6)	Ce1—O1W—H1WB	132 (2)
C7—O1—Ce1	128.03 (16)	H1WA—O1W—H1WB	108 (2)
C13—O5—Ce1	127.41 (16)	Ce1—O2W—H2WA	128 (2)
C14—O7—H7	109 (3)	Ce1—O2W—H2WB	122 (2)
C1—N1—C5	118.0 (2)	H2WA—O2W—H2WB	109 (2)
C1—N1—Ce1	125.81 (17)	Ce1—O3W—H3WA	117 (2)
C5—N1—Ce1	116.15 (16)	Ce1—O3W—H3WB	125 (2)
C12—N2—C8	118.1 (2)	H3WA—O3W—H3WB	109 (2)
C12—N2—Ce1	124.06 (16)	Ce1—O4W—H4WA	122 (2)
C8—N2—Ce1	117.68 (16)	Ce1—O4W—H4WB	124 (2)
N1—C1—C2	123.2 (2)	H4WA—O4W—H4WB	109 (2)
N1—C1—H1A	118.4	Ce1—O5W—H5WA	120 (2)
C2—C1—H1A	118.4	Ce1—O5W—H5WB	121 (2)
C3—C2—C1	117.7 (2)	H5WA—O5W—H5WB	112 (2)
C3—C2—C6	121.5 (2)	H6WA—O6W—H6WB	112 (2)
C1—C2—C6	120.8 (2)	H7WA—O7W—H7WB	111 (2)
C2—C3—C4	119.7 (3)	H8WA—O8W—H8WB	112 (2)
C2—C3—H3A	120.1	H9WA—O9W—H9WB	111 (2)

### Hydrogen-bond geometry (Å, °)

**Table:**

*D*—H···*A*	*D*—H	H···*A*	*D*···*A*	*D*—H···*A*
O1W—H1WA···O8W	0.83 (1)	1.94 (1)	2.747 (3)	165 (3)
O1W—H1WB···O3^i^	0.83 (1)	1.81 (1)	2.630 (3)	170 (3)
O2W—H2WA···O6W	0.83 (1)	1.96 (1)	2.779 (3)	167 (3)
O2W—H2WB···O6W^ii^	0.83 (1)	2.11 (2)	2.898 (3)	159 (3)
O3W—H3WA···O7W	0.83 (1)	1.98 (1)	2.816 (3)	177 (3)
O3W—H3WB···O4^iii^	0.83 (1)	2.01 (1)	2.823 (3)	165 (4)
O4W—H4WA···O7W^iv^	0.83 (1)	1.86 (1)	2.687 (3)	175 (3)
O4W—H4WB···O9W	0.83 (1)	1.88 (1)	2.689 (3)	166 (3)
O5W—H5WA···O8^v^	0.83 (1)	1.96 (1)	2.789 (3)	175 (3)
O5W—H5WB···O2^vi^	0.83 (1)	2.01 (1)	2.821 (3)	167 (4)
O6W—H6WA···O2^vi^	0.83 (1)	2.04 (2)	2.823 (3)	157 (3)
O6W—H6WB···O3^vii^	0.83 (1)	1.97 (3)	2.698 (3)	146 (4)
O7W—H7WA···O8W^vii^	0.83 (1)	1.94 (1)	2.747 (4)	164 (3)
O7W—H7WB···O6^viii^	0.83 (1)	2.28 (2)	3.054 (3)	157 (5)
O7W—H7WB···O8^v^	0.83 (1)	2.45 (4)	2.898 (3)	115 (3)
O8W—H8WA···O2^ix^	0.82 (1)	2.00 (2)	2.765 (3)	155 (3)
O8W—H8WA···O1^ix^	0.82 (1)	2.64 (2)	3.364 (3)	148 (3)
O8W—H8WB···O7^ix^	0.83 (1)	2.12 (2)	2.901 (3)	158 (4)
O9W—H9WA···O4^x^	0.83 (1)	1.94 (1)	2.750 (3)	167 (4)
O9W—H9WB···O5W^xi^	0.82 (1)	2.33 (2)	3.087 (4)	154 (4)
O7—H7···O6^xii^	0.82 (1)	1.71 (1)	2.526 (3)	173 (5)

Symmetry codes: (i) −*x*+2, −*y*, −*z*+1; (ii) −*x*+3/2, −*y*−1/2, −*z*+1; (iii) −*x*+2, −*y*−1, −*z*+1; (iv) *x*+1/2, *y*+1/2, *z*; (v) −*x*+3/2, *y*+1/2, −*z*+1/2; (vi) *x*−1/2, *y*+1/2, *z*; (vii) *x*−1/2, *y*−1/2, *z*; (viii) −*x*+3/2, *y*−1/2, −*z*+1/2; (ix) *x*, *y*+1, *z*; (x) −*x*+5/2, −*y*−1/2, −*z*+1; (xi) *x*+1/2, *y*−1/2, *z*; (xii) *x*, *y*−1, *z*.
